# Gold Standard Cholera Diagnostics Are Tarnished by Lytic Bacteriophage and Antibiotics

**DOI:** 10.1128/JCM.00412-20

**Published:** 2020-08-24

**Authors:** E. J. Nelson, J. A. Grembi, D. L. Chao, J. R. Andrews, L. Alexandrova, P. H. Rodriguez, V. V. Ramachandran, M. A. Sayeed, J. F. Wamala, A. K. Debes, D. A. Sack, A. J. Hryckowian, F. Haque, S. Khatun, M. Rahman, A. Chien, A. M. Spormann, G. K. Schoolnik

**Affiliations:** a Departments of Pediatrics and Environmental and Global Health, University of Florida, Gainesville, Florida, USA; b Department of Pediatrics, School of Medicine, Stanford University, Stanford, California, USA; c Department of Civil and Environmental Engineering, Stanford University, Stanford, California, USA; d Institute for Disease Modeling, Bellevue, Washington, USA; e Department of Medicine, School of Medicine, Stanford University, Stanford, California, USA; f Vincent Coates Foundation Mass Spectrometry Laboratory, Stanford University, Stanford, California, USA; g Country Preparedness and IHR (CPI), World Health Organization (South Sudan), Juba, South Sudan; h Johns Hopkins Bloomberg School of Public Health, Johns Hopkins University, Baltimore, Maryland, USA; i Department of Microbiology, School of Medicine, Stanford University, Stanford, California, USA; j Institute of Epidemiology, Disease Control and Research, Ministry of Health and Family Welfare, Government of Bangladesh, Dhaka, Bangladesh; Johns Hopkins University School of Medicine

**Keywords:** ICP1, ICP2, ICP3, RDT, bacteriophage, cholera, cholerae, outbreak, vibrio, vibriophage

## Abstract

A fundamental, clinical, and scientific concern is how lytic bacteriophage, as well as antibiotics, impact diagnostic positivity. Cholera was chosen as a model disease to investigate this important question, because cholera outbreaks enable large enrollment, field methods are well established, and the predatory relationship between lytic bacteriophage and the etiologic agent Vibrio cholerae share commonalities across bacterial taxa. Patients with diarrheal disease were enrolled at two remote hospitals in Bangladesh. Diagnostic performance was assessed as a function of lytic bacteriophage detection and exposure to the first-line antibiotic azithromycin, detected in stool samples by mass spectrometry. Among diarrheal samples positive by nanoliter quantitative PCR (qPCR) for V. cholerae (*n* = 78/849), the odds that a rapid diagnostic test (RDT) or qPCR was positive was reduced by 89% (odds ratio [OR], 0.108; 95% confidence interval [CI], 0.002 to 0.872) and 87% (OR, 0.130; 95% CI, 0.022 to 0.649), respectively, when lytic bacteriophage were detected. The odds that an RDT or qPCR was positive was reduced by more than 99% (OR, 0.00; 95% CI, 0.00 to 0.28) and 89% (OR, 0.11; 95% CI, 0.03 to 0.44), respectively, when azithromycin was detected. Analysis of additional samples from South Sudan found similar phage effects on RDTs; antibiotics were not assayed. Cholera burden estimates may improve by accommodating for the negative effects of lytic bacteriophage and antibiotic exposure on diagnostic positivity. One accommodation is using bacteriophage detection as a proxy for pathogen detection. These findings have relevance for other diagnostic settings where bacterial pathogens are vulnerable to lytic bacteriophage predation.

## Abstract (precis)

A fundamental, clinical, and scientific concern is how lytic bacteriophage, as well as antibiotics, impact diagnostic positivity. Cholera was chosen as a model disease to investigate this important question, because cholera outbreaks enable large enrollment, field methods are well established, and the predatory relationship between lytic bacteriophage and the etiologic agent Vibrio cholerae share commonalities across bacterial taxa. Patients with diarrheal disease were enrolled at two remote hospitals in Bangladesh.

## INTRODUCTION

There are approximately 4.5 billion diarrheal disease cases per year (1). While the 2 to 4 million cases of cholera that occur annually represent a small fraction of the total cases (2), cholera inflicts a high morbidity and mortality on populations with extreme poverty. Outbreaks begin when immunologically susceptible human hosts are exposed to the Gram-negative pathogen Vibrio cholerae (O1 and O139 serogroups) from contaminated food or water (3). Before modern rehydration regimens, mortality rates rose above 20% (4) from acute secretory diarrhea resulting from the action of cholera toxin (3). For cases treated with oral or intravenous rehydration, mortality rates decrease to less than one percent (5, 6). Antibiotics are recommended for cholera patients with moderate to severe dehydration (7, 8), but in practice most cholera patients are likely ordered antibiotics (27, 36, 44). Asymptomatic cases are detected by a rise in antibody titer but negative stool studies, typically by microbial culture (9). V. cholerae is shed from the human host with increased infectivity (10, 11). This hyperinfectivity is suggested to drive the exponential phase of outbreaks (12).

Patients often shed V. cholerae-specific lytic bacteriophages (ICP1, ICP2, and ICP3). The receptor for ICP1 is the O-antigen lipopolysaccharide (13–15), and the ICP2 receptor is OmpU, which is regulated by ToxR; removal of either protein impedes ICP2 (13, 16). The receptor for ICP3 is assumed to be the O-antigen lipopolysaccharide (17). While the host range of ICP1 is O1 V. cholerae, ICP2 and ICP3 can infect non-O1 V. cholerae (16). These vibriophages are proposed to quench outbreaks based on data that a higher percentage of patients shed vibriophages during the collapse of an outbreak (18–21). Vibriophages are detected in aquatic settings with correlates to outbreaks (19, 22), and ICP1 has been shown to maintain infectivity in environmental water (23).

Diagnostically, culture and PCR are the best available gold standards for the detection of V. cholerae (24, 25). Alternative methods include direct immunofluorescence microscopy for the O-antigen polysaccharide (OPS) (26), rapid diagnostic tests (RDTs) that rely on OPS-specific antibodies, and, recently, nanoliter quantitative PCR (nl-qPCR) (27, 28); nl-qPCR uses standard quantitative PCR chemistry in nanoliter reaction volumes that are loaded robotically onto chips with 5,000 wells (TaKaRa Bio USA; formerly WaferGen, Inc.). RDTs are intended to be durable in field settings where clinical laboratories are not available but diagnostic information is vital to outbreak response (25). Commercialized RDTs for cholera typically consist of a lateral-flow device (dipstick). One end of the RDT is placed in the test material that also contains a pad with mobile gold-labeled monoclonal antibody for the target pathogen. The gold-labeled antibody bound to antigen is drawn vertically up the RDT by capillary action past a test line with unlabeled monoclonal antibody for the target pathogen and then a control line with a goat anti-mouse antibody that binds the nonvariable region of the monoclonal antibody (29).

The rationale for this study was based on the recognition that cholera RDTs have limited adoption because of variable performance for unknown reasons (25, 30–33); immediately testing stool samples demonstrated broad sensitivities (58 to 100%) and specificities (71 to 100%). A modified method that enriches for V. cholerae in alkaline peptone water (APW) to increase specificity to 91 to 99% is associated with a decrease in sensitivity (30, 31, 34). Both lytic bacteriophage and antibiotics have been postulated to impact diagnostics (33). Developing an RDT that has both high sensitivity and specificity is a formidable task. One of the most commonly used RDTs is a lateral-flow RDT for group A *Streptococcus* (Gram positive). This RDT has a sensitivity of 86% and specificity of 95% (35). Clinically, this means 14 out of 100 patients may have streptococcal pharyngitis but might be misdiagnosed and, therefore, not receive the indicated antibiotic. An important question that has generalizability is if antibiotics or lytic bacteriophage affect the results in these 14 patients.

Using cholera as a model, we tested the hypothesis that lytic bacteriophage, and antibiotics, negatively impact diagnostics within the confines of a previously published clinical study (36). In brief, the study was conducted from September to December 2015 at the district hospital, and a subdistrict hospital, in the remote northern district of Netrokona, Bangladesh, which is prone to seasonal cholera outbreaks. Inclusion criteria were patients at least 2 months old and presenting with acute (<7 days) diarrhea (>3 loose stools in the 24 h prior to admission) without complications. Additional analyses were performed on samples from South Sudan to broaden the experimental scope of this study.

## MATERIALS AND METHODS

### Participants.

This study was conducted within the confines of previously published studies in Bangladesh (36) and South Sudan (37). Ethical approvals were obtained for the Bangladesh study at the Institutional Review Boards (IRBs) of Stanford University School of Medicine and the Institute of Epidemiology, Disease Control and Research, Bangladesh Ministry of Health and Family Welfare (36), and for the South Sudan study at the IRBs of Johns Hopkins Bloomberg School of Public Health and the South Sudan Ministry of Health, Directorate of Monitoring, Evaluation, and Research (37). Written informed consent/assent was obtained from participants and/or guardians of participants.

### Study design.

In Bangladesh, inclusion criteria were patients at least 2 months of age presenting with acute (<7 days) diarrhea (>3 loose stools in the prior 24 h) without clinical complications. Sample collection occurred from September to December 2015 at the district hospital and a subdistrict hospital in the remote northern district of Netrokona, which is prone to seasonal cholera outbreaks. In South Sudan, inclusion criteria were patients presenting at a cholera treatment center in Juba who were at least 6 months old and had diarrhea (>3 loose stools in the prior 24 h). Samples were collected from August to September 2015. There was no history of cholera vaccination at the Bangladesh and South Sudan sites.

### Laboratory procedures.

For samples collected in Bangladesh, the methods have been previously described (27). In brief, the first stool sample voided was collected immediately after admission to avoid exposure to hospital-administered antibiotics. The supernatants from V. cholerae-positive stools were tested for antibiotic exposure using a liquid chromatography-mass spectrometry (LC-MS) protocol for a 1100 series high-performance liquid chromatograph (Agilent Technologies) integrated with an LTQ XL ion trap mass spectrometer (Thermo Fisher Scientific) (27). The stool samples were tested by a dipstick RDT (Crystal VC, Span Diagnostics) after enrichment in APW for 6 h or overnight (36). The first and last samples collected per day were stored in Cary-Blair medium (4°C) for culture at a central reference laboratory in Dhaka (icddr,b); samples were stored for up to 1 month. Aliquots (500 μl) from all patients were stored in 1.3 ml RNA*later* (Invitrogen).

For Bangladesh samples, stools suspended in RNA*later* were centrifuged to obtain pellets for DNA extraction using the MoBio Power Soil 96-well plate system (Qiagen; formerly PowerSoil). DNA extracts were screened in technical replicates for V. cholerae by qPCR in a 384-well LightCycler (Roche) using *tcpA*^set1^ primers (see Table S1 in the supplemental material) (27). Samples that had threshold cycle (*C_T_*) values of less than 25 were defined as positive. Samples with *C_T_* values from 25 to 31 were evaluated for the second target of *ompW* by PCR and gel electrophoresis, given that this *C_T_* value range is vulnerable to false positives and negatives (8). In parallel, nl-qPCR was performed in technical replicates with *tcpA*^set1^ primers and additional targets (27, 28). SYBR Green master mix (Sigma-Aldrich) was used for both qPCR and nl-qPCR; however, there was 1.8-fold more DNA in nl-qPCRs. Cycle threshold values for positivity for qPCR and nl-qPCR were 29 and 28, respectively. 16S rRNA gene analysis utilized previously published methods and data (27) on nl-qPCR V. cholerae-positive samples for *tcpA* (Table S1). Lytic vibriophages ICP1, ICP2, and ICP3 were detected by PCR (Table S1). For samples collected in South Sudan, analyses for V. cholerae have been previously described on DNA extracted from dried stool spots (37). In addition, the extracts were analyzed by PCR for ICP1 and ICP3 (ICP2 PCR technically failed; Table S1).

Direct immunofluorescence was performed as previously described on planktonic cells from RNA*later*-preserved stool samples (38). The planktonic cell fraction was obtained by a 15-s centrifugation at 100 × *g* to remove sediment from 500 μl of sample followed by one phosphate-buffered saline (PBS) wash, pelleting the supernatant fraction, and resuspension of the pellet in 500 μl of PBS with 3.7% formalin. Mock positive-control stool samples were used for molecular and microscopy assays that consisted of V. cholerae set to concentrations relative to cholera stool (5e8 CFU/ml and 1e8 CFU/ml) in 500 ml normal saline plus 1.3 ml RNA*later* (ratio used in stool storage).

### Statistical analysis.

Latent class modeling was used to estimate sensitivities and specificities of each diagnostic (39). For prior information, the assumptions for sensitivities were the same for RDT, qPCR, nl-qPCR, and culture (50 to 100%). Assumptions for specificities were 50 to 100% for RDT, 90 to 100% for qPCR and nl-qPCR, and 99 to 100% for culture (24). Gibbs sampling with 100,000 iterations was used to generate posterior estimates with 95% confidence intervals (CI). Fisher's exact test was used to evaluate associations between diagnostic type and detection of lytic bacteriophage/azithromycin. Both sample odds ratios (OR) and estimated sample odds ratios with a conditional maximum likelihood estimate were computed. A two-sample Wilcoxon test was used to compare *C_T_* values between diagnostic positive and negative samples among samples positive for V. cholerae by nl-qPCR *C_T_*. Comparison of microbiota (16S rRNA gene analysis) by diagnostic result and exposure among nl-qPCR-positive samples was conducted by permutational multivariate analysis of variance (PERMANOVA) as previously described (27). Missingness in the data set is designated NA and is restricted to laboratory results. Statistical analyses were completed in GraphPad Prism 8.0.1 and R v3.4.1/RStudio v1.1.0153 (40).

### Data availability.

Data analyzed in the manuscript are available in the online supplemental material.

## RESULTS

### Sensitivity and specificity estimates by latent class modeling.

**FIG 1:**
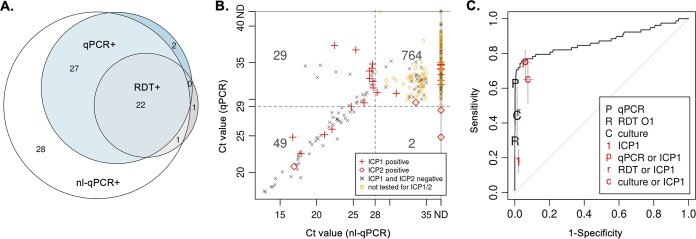
Diagnostic comparison of Bangladesh stool samples identified as positive for V. cholerae by at least one modality (RDT, qPCR, and nl-qPCR; *N* = 78). (A) Venn diagram of diagnostic positivity for qPCR, nl-qPCR, and RDT; area within each circle is relative to the degree of positivity. (B) Comparison *C_T_* values between qPCR and nl-qPCR analysis with ICP1 and ICP2 metadata; horizontal and vertical dotted lines depict thresholds of positivity for each test. ND, not detected. (C) Receiver operator characteristic (ROC) curve. Estimates of the sensitivity and 1-specificity of combining diagnostics are defined in the key, and vertical bars from each symbol depict the 95% CI; these data are available in tabular format in Table S4.

In Bangladesh, stool samples were collected from 881 of 961 enrolled patients. Among samples tested by RDT, qPCR, and nl-qPCR, the distribution of diagnostic positivity is provided (Fig. 1A and B). The sensitivities and specificities of each diagnostic were estimated using a Bayesian latent class modeling framework, which enables estimation of diagnostic accuracy in the absence of a perfect reference standard by integrating data from multiple tests (39). Estimates for sensitivity of RDT, qPCR, and nl-qPCR were 31.5% (95% CI, 21.5 to 43.7), 64.1% (95% CI, 50.7 to 80.2), and 97.6% (95% CI, 89.0 to 100.0), respectively. The specificities were 99.6% (95% CI, 99.0 to 99.9), 99.9% (95% CI, 99.7 to 100.0), and 99.6% (95% CI, 98.3 to 100.0), respectively. Among the subset of samples randomly chosen for culture (16 positive out of 251), sensitivity was 57.1% (40.4 to 73.2) and specificity was 99.7 (99.3 to 99.9). Based on these results, nl-qPCR was selected as the best available reference standard for subsequent analysis. The receiver operator curve (ROC) is presented (Fig. 1C).

### Impact of lytic phage on diagnostic positivity.

Among V. cholerae-positive samples by nl-qPCR in Bangladesh, 19.2% (15/78) and 1.3% (1/78) were positive for ICP1 and ICP2, respectively; ICP3 was not detected. Of 180 random samples negative by nl-qPCR, qPCR, and RDT, two patients had ICP1 (one was culture positive) and one had ICP2. Among V. cholerae-positive samples by nl-qPCR that lacked azithromycin, vibriophages (ICP1 and ICP2) were negatively associated with diagnostic positivity by RDT (OR, 0.11; 95% CI, 0.002 to 0.87), qPCR (OR, 0.13; 95% CI, 0.02 to 0.65), and direct immunofluorescence microscopy (38) (OR, 0.18; 95% CI, 0.02 to 1.031; Table 1). Frequencies of vibriophage detection were different between study sites (Fischer’s exact test; *P* = 0.033).

**TABLE 1:** Lytic phage negatively impact diagnostic positivity (azithromycin excluded)

Dx^*g*^	*N*^*a*^	Dx positive [% (no. positive/total no.)] by phage exposure		OR^*b*^	OR__MLE_^*c*^	CI^*c*^	*P* value^*d*^
		Exposed	Unexposed				
RDT	56	9 (1/11)	49 (22/45)	0.105	0.108	0.002–0.872	0.019
qPCR	56	36 (4/11)	83 (37/45)	0.124	0.130	0.022–0.649	0.005
Microscopy^*e*^	52	20 (2/10)	60 (25/42)	0.170	0.176	0.016–1.031	0.036
Culture^*f*^	22	0 (0/0)	59 (13/22)				

a Number of V. cholerae-positive samples by nl-qPCR without azithromycin detected in the stool by mass spectrometry.

b OR, sample odds ratio.

c Estimated odds ratio with conditional maximum likelihood estimate (MLE); CI, 95% confidence interval.

d Fisher's exact test.

e Indeterminant samples were considered negative; limit of detection was 100 to 1,000 CFU/ml.

f Insufficient samples with phage for statistical analysis.

g Dx, diagnostic.

Diarrheal samples from South Sudan were analyzed to increase generalizability (37). ICP1 was detected in 10.2% of enriched samples (*n* = 10/98) independent of V. cholerae detection by PCR. Among samples positive for V. cholerae by PCR, ICP1 was detected in 24% of samples (*n* = 7/29; see Table S2 in the supplemental material). Among samples negative for V. cholerae by PCR, ICP1 was detected in 4.3% of samples (*n* = 3/69); two samples were RDT negative, and one was weakly RDT positive. ICP1 was negatively associated with RDT positivity after enrichment (OR, 0.00; 95% CI, 0.00 to 0.64, *P* = 0.010; Table S2); a statistically significant difference was not observed for unenriched samples. ICP3 was not identified. There were insufficient samples with bacteriophage detected to assess bacteriophage impact on culture positivity.

### Impact of azithromycin on diagnostic positivity.

Among Bangladesh samples positive by nl-qPCR but negative for bacteriophage, azithromycin was negatively associated with diagnostic positivity by RDT (OR, 0.00; 95% CI, 0.00 to 0.28) and qPCR (OR, 0.11; 95% CI, 0.03 to 0.44) but not by direct fluorescence microscopy (OR, 0.54; 95% CI, 0.14 to 1.97) (Table 2). Azithromycin was negatively associated with culture positivity (OR, 0.00; 95% CI, 0.00 to 0.997) (Table 2).

**TABLE 2:** Azithromycin negatively impacts diagnostic positivity (phage excluded)

Dx^*g*^	N^*a*^	Dx positive [% (no. positive/total no.)] by phage exposure:		OR^*b*^	OR__MLE_^*c*^	CI^*c*^	*P* value^*d*^
		Exposed	Unexposed				
RDT	63	0 (0/18)	49 (22/45)	0.000	0.000	0.000–0.282	<0.001
qPCR	63	33 (6/18)	82 (37/45)	0.108	0.113	0.026–0.437	<0.001
Microscopy^*e*^	58	44 (7/16)	60 (25/42)	0.529	0.535	0.139–1.973	0.378
Culture^*f*^	27	0 (0/5)	59 (13/22)	0.000	0.000	0.000–0.997	0.041

a Number of V. cholerae-positive samples by nl-qPCR without lytic bacteriophage (ICP1, ICP2, and ICP3) detected in the stool by PCR.

b OR, sample odds ratio.

c Estimated odds ratio with conditional maximum likelihood estimate (MLE); CI, 95% confidence interval.

d Fisher's exact test.

e Indeterminant samples were considered negative; limit of detection 100 to 1,000 CFU/ml.

f Insufficient samples with phage for statistical analysis.

g Dx, diagnostic.

### Absolute and relative V. cholerae concentration.

Absolute and relative V. cholerae concentration was assessed by nl-qPCR and 16S rRNA gene analysis, respectively. Among nl-qPCR-positive samples, there was a significant inverse relationship between diagnostic positivity and V. cholerae concentration (Table S3). With no exclusions, fold differences between positive and negative samples ranged from 21-fold (culture) to 79-fold (qPCR). The one exception was that phage exposure (azithromycin samples excluded) did not associate with a significant difference in the nl-qPCR *C_T_* values between culture-positive (*n* = 13; *C_T_* , 19.4; 95% CI, 14.3 to 22.0) and -negative samples (*n* = 9; *C_T_*, 20.8; 95% CI, 17.6 to 25.9; *P* = 0.186). Statistically significant differences in microbiota (16S rRNA gene) were observed between RDT-positive and -negative stools with stratifications for bacteriophage (Fig. S2A) and azithromycin (Fig. S2B).

## DISCUSSION

This study investigated the potential vulnerability diagnostics have when bacterial targets are exposed to lytic bacteriophage predation or antibiotics. Using cholera as a model system and nl-qPCR as a reference standard for V. cholerae, we found that the odds of an RDT, qPCR, and microscopy diagnostic testing positive were reduced by more than 83% when lytic bacteriophage were present. Similarly, the odds of an RDT, qPCR, and culture testing positive were reduced by more than 89% when the first-line antibiotic azithromycin was detected in stool by mass spectrometry. These results expose a vulnerability of gold-standard diagnostics that clinicians and scientists have feared but lacked sufficient data to take evidence-based action.

We reason that the low inflection point in the ROC at approximately 0.7 sensitivity is multifactorial (Fig. 1C). We explored the effect on sensitivity and specificity of adding ICP1 detection as a proxy for V. cholerae detection (Fig. 1C; see also Table S4 in the supplemental material). qPCR, culture, and RDT results moderately improved. The effects of lytic bacteriophage, antibiotics, and host antimicrobial factors on diagnostic positivity are likely additive, especially given that these diagnostics target different biologic mechanisms. How duration of illness and severity of disease serve as determinants of diagnostic positivity remain unknown. Time-series analyses of cholera patients with lytic bacteriophage coinfection and defined antimicrobial administration are needed to further these lines of inquiry. These studies would be strengthened by enrollment of household contacts and healthy neighbor households, as well as testing their water sources, to collectively determine rates of symptomatic and asymptomatic V. cholerae infection and for detection of vibriophage inside and outside the human host.

These findings should be viewed within the context of the limitations of the study. The procedures were chosen for feasibility at remote field sites. The remote locations delayed transport and culture up to 1 month, which can be detrimental to culture efficiency (41) and precluded plaque assays by soft-agar overlay with V. cholerae exposed to filtered stool supernatant. The higher detection rate of nl-qPCR than that of qPCR was multifactorial, including the 1.8-fold difference in DNA. The positive nl-qPCR samples that were negative by qPCR and negative by *ompW* were unlikely to be false positives, because *Vibrio* spp. were detected by 16S rRNA gene analysis in all samples that did not have lytic vibriophage (*n* = 13/13); those samples with vibriophage had DNA of insufficient quality and/or quantity to yield a 16S rRNA gene result (*n* = 7/7). Among nl-qPCR-positive and qPCR-negative samples, PCR detection for *tpcA* correlated with PCR detection of *ctxA* (cholera toxin; *n* = 5/5; Table S1). These data, paired with serologic results that found only O1 V. cholerae, make the possibility of confounding from non-O1 V. cholerae unlikely. Despite these limitations, the discovery that lytic bacteriophage negatively impacts diagnostics by 5- to 10-fold, even to the point that samples will test positive for bacteriophage and negative for the pathogen, has broad significance. One explanation is lytic bacteriophage and antibiotics inhibit bacterial growth below the diagnostic limits of detection. Alternatively, bacteriophage nucleases, or host nucleases responding to bacteriophage infection, may differentially digest host chromosomal DNA to the point that PCR fails (42, 43).

### Conclusions.

Within the cholera field, this study suggests that an updated approach is needed to estimate cholera burden, especially in the latter phases of outbreaks when rates of concurrent lytic bacteriophage predation are likely higher (19, 20). This may require an approach that includes lytic bacteriophage detection as a proxy for pathogen detection and a deemphasis on diagnostic results with known antibiotic exposure. Outside the cholera field, these data serve as a call to action to survey for lytic bacteriophage when bacterial diagnostics have inconsistent performance. These efforts may justify a new line of diagnostic development that targets both the prey (pathogen) and predator (bacteriophage) and scientific inquiry into the underlying mechanisms of action and spatial/temporal relationship of lytic bacteriophage and their host pathogen.

## Supplementary Material

Supplemental file 1

Supplemental file 2
