# Supplementary material for: Gold Standard Cholera Diagnostics Are Tarnished by Lytic Bacteriophage and Antibiotics
Source: J Clin Microbiol. 2020 Aug 24;58(9):e00412-20. doi: 10.1128/JCM.00412-20 (PMC7448619; doi:10.1128/JCM.00412-20)
Supplement: Supplemental file 1 [file JCM.00412-20-s0002.pdf]

SUPPLEMENTAL MATERIAL

Table of contents

1. **Fig. S1.** Diagnostic positivity and ICP1 detection in South Sudan and Bangladesh
2. **Fig. S2.** Microbiota distribution as a function of bacteriophage and azithromycin detection
3. **Table S1.** Reagents
4. **Table S2.** South Sudan: Impact of bacteriophage ICP1 on cholera RDT positivity
5. **Table S3.** Fold-differences in DNA concentration among V. cholerae positive samples by nl qPCR
6. **Table S4:** Estimated sensitivity and specificity of theoretical diagnostic modalities
7. **References**
8. **Dataset**

Figure S1

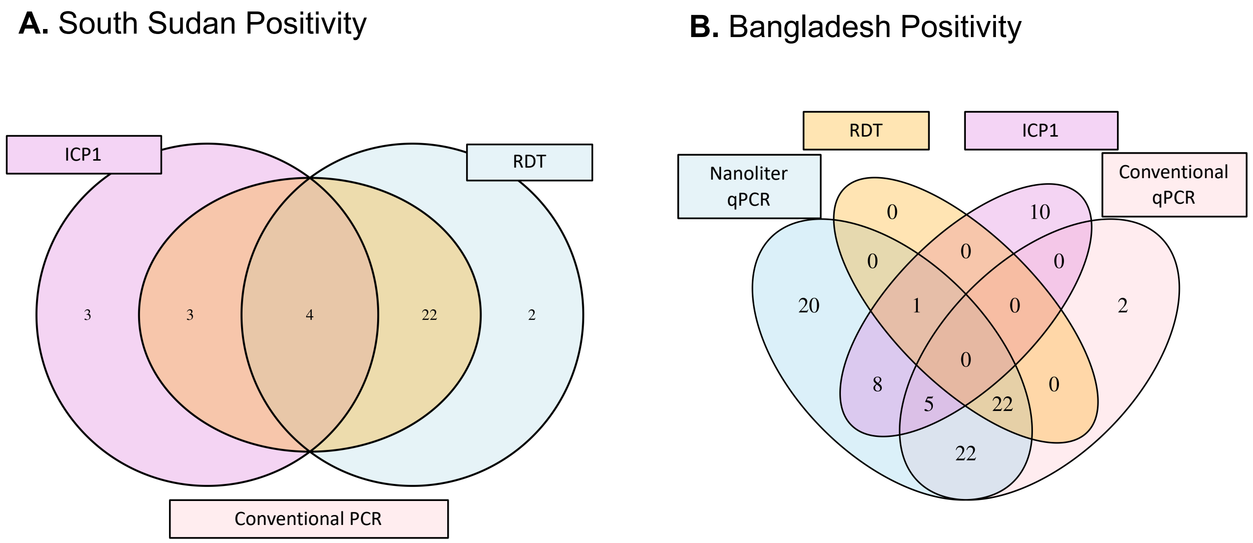

Fig. S1. Venn diagrams comparing diagnostic positivity and ICP1 detection in the libraries from South Sudan **(A)** and Bangladesh **(B).** RDT= rapid diagnostic test. Both settings used the Crystal VC test with enrichment. Data presented from S. Sudan are based on PCR studies performed at Institute Pasteur (Table S2) (11).

Figure S2

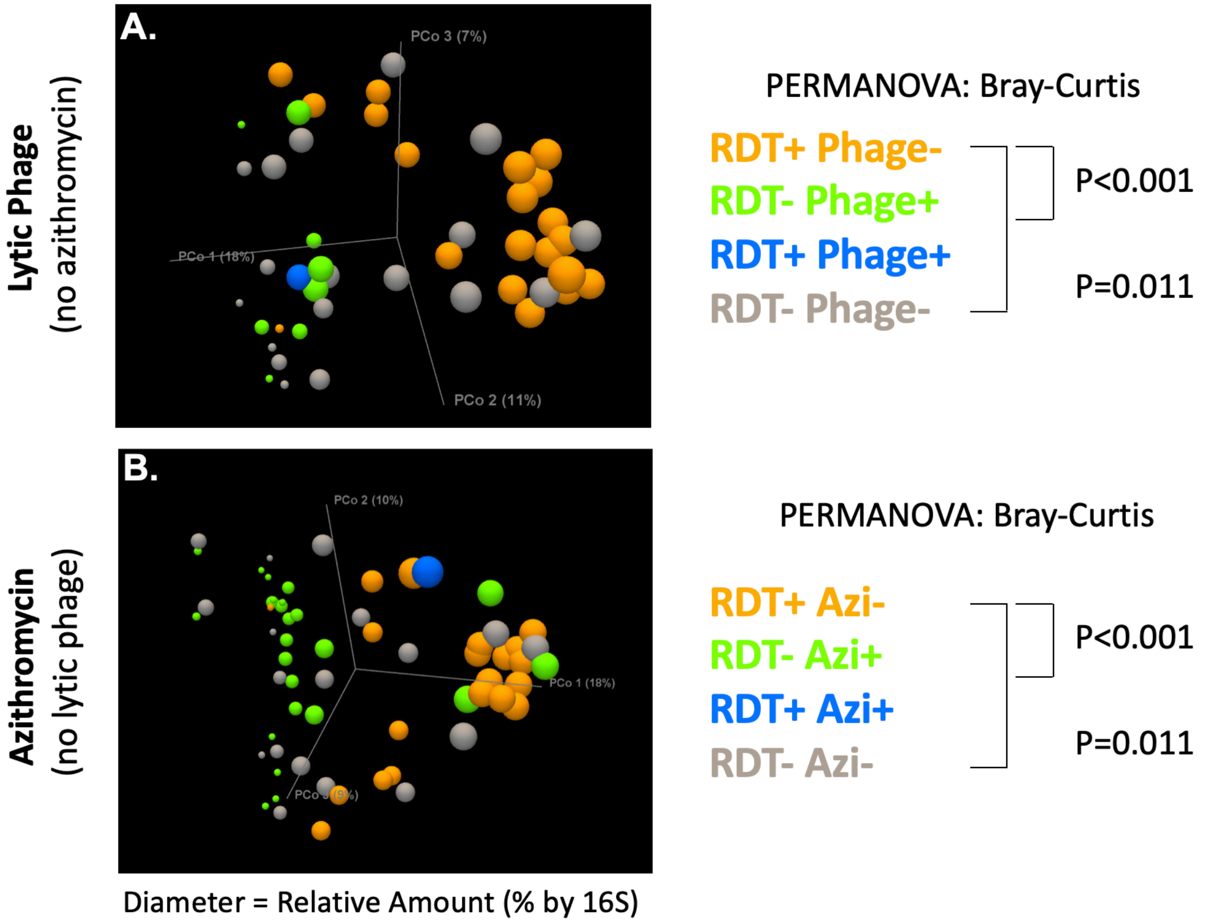

Fig. S2**.** Principal component analysis of 16S rDNA analysis of *V. cholerae* positive samples by nl-qPCR analysis as previously described (1). **A.** Among samples without azithromycin detection, data are color-coded based on RDT and phage positivity (right). **B.** Among samples without bacteriophage detection, data are color-coded based on RDT and azithromycin positivity (right). PC1 is oriented horizontally and the icon size is set to the relative percentage of *V. cholerae* detected in the microbiota (0-25%, 26-50%, 51-75%, 76-100%). For both the upper and lower panels, statistically significant differences between groups were detected by PERMANOVA (Bray-Curtis) (1). *V. cholerae* positivity is defined by nl-qPCR positivity with either *tcpA* primer sets to be consistent with prior analytic approach(1).

Table S1. Reagents

**Reagent**			
**Bacterium**	**Strain**	**Description**	**Reference**
V. cholerae	E7946	O1 serogroup, isolated from Bahrain*, SmR	Mekalanos (2)
V. cholerae	ENV2	Non-O1, from Haiti*, SmS	Rahman et al. (3)
V. cholerae	VC037; 280 NAG	Non-O1, from India*, ATCC25872, SmR	Felsenfeld et al. (4)
**PCR target**	**Primer name**	**Sequence 5’ – 3’**	
ctxA	ctxA_F	CTCAGACGGGATTTGTTAGGCACG	Hoshino et al. (5)
	ctxA_R	TCTATCTCTGTAGCCCCTATTACG	
ompW	ompW_F	CACCAAGAAGGTGACTTTATTGTG	Nandi et al. (6)
	ompW_R	GAACTTATAACCACCCGCG	
tcpA	tcpA^{set1}_F	ACTAAGGCTGCGCAAAATCT	Grembi & Spormann(7)
	tcpA^{set1}_R	GCCTCATCAGCTGAAACCTT	
tcpA	tcpA^{set2}_F	ACACGATAAGAAAACCGGTCA	Grembi & Spormann(7)
	tcpA^{set2}_R	GCCTTGGTCATATTCTGCGA	
ICP1	ICP1gp58F	AACGCTGCTTTTCCTTTTGA	Seed et al. (8)
	ICP1gp58R	CCCAGCATTGAGGACACTT	
ICP2	ICP2_4F	CGCTAGTTCTGGCAGTGA GT	Alexandrova et al.(1)
	ICP2_4R	TCCGTTCCAGTTCCAACAGG	
ICP2	ICP2_24F	AGAAGTCGCAAACGGGGTAC	Alexandrova et al.(1)
	ICP2_24R	AACGTGGTTCTCGTGAGTGG	
ICP3	ICP3gp5F	ATTGTCGAGTGGGACAAAGG	Seed et al. (8)
	ICP3gp5R	ACCAACTCGACGCATAGCTT	
16S rDNA**	Maeda_1048_1067_F	GTGGSTGCAYGGYTGTCGTCA	Maeda et al. (9)
	Maeda_1175_1194_R	ACGTCRTCCMCACCTTCCTC	
16S rDNA**	27F_Miseq	AATGATACGGCGACCACCGAGATCTACA C*TATGGTAATT*ccAGMGTTYGATYMTGG CTCAG	Chung et al. (10)
	338rcbc1	CAAGCAGAAGACGGCATACGAGAT **ACGAGACTGATT***AGTCAGTCAG*aaGCTG CCTCCCGTAGGAGT	Chung et al. (10)

* Strain used as a control strain for testing specificity of *tcpA* primers set1 and set2.
** 16S rDNA primer pair used for nanoliter qPCR. Degenerate primers are coded per standard convention (http://arep.med.harvard.edu/labgc/adnan/projects/Utilities/revcomp.html). Example of 16S rDNA primer pair used for microbiome analysis. Degenerate primers are coded per standard convention. Structure of forward primer: (i) 5' Illumina adapter, (ii) *Forward primer pad*, (iii) Forward primer linker (lower case), (iv) Forward primer. Structure of reverse primer example: (i) Reverse complement of 3′ Illumina adapter (underlined), (ii) Golay barcode (bold text), (iii) Reverse primer pad (italics), (iv) Reverse primer linker (lower case), (v) Reverse primer.

Table S2. South Sudan: Impact of bacteriophage ICP1 on cholera RDT positivity

		Diagnostic Positive		Diagnostic Negative				
Diagnostic Test	Total	Phage Pos	Phage Neg	Phage Pos	Phage Neg	$OR_{\_MLE}$[a]	CI[a]	P[b]
Among VC PCR positive (Hopkins)[c]								
RDT (Direct)[d]	32	7	26	1	0	---	---	---
RDT (Enriched)	34	5	25	3	1	0.075	0.001-1.137	0.033
Among VC PCR positive (Pasteur)[c]								
RDT (Direct)[d]	29	6	22	1	0	---	---	---
RDT (Enriched)	29	4	22	3	0	0.000	0.000-0.641	0.010

[a] Estimated odds ratio with conditional Maximum Likelihood Estimate (MLE); CI = 95th confidence interval.
[b] Fisher's exact test.
[c] Conventional PCR was performed by Johns Hopkins University and Institute Pasteur for cross validation (11). 'Direct' = tested directly from stool; 'Enriched' = grown in selective media (APW) prior to testing. Values reported in the main text and Figure S1 are derived from the Institute Pasteur data set; 'strong positive' and 'weak positive' RDT values were analyzed as equivalent.
[d] Insufficient sample size for statistical analysis.

Table S3. Fold-differences in target DNA detection among *V. cholerae* positive samples

Diagnostic Test	Total[1]	Diagnostic Positive Median nl-qPCR Ct (n; IQR)	Diagnostic Negative Median nl-qPCR Ct (n; IQR)	Fold-difference[4]	p[2]
No exclusion					
RDT	78	19.0 (23; 16.5-21.7)	24.4 (55; 21.1-27.7)	42	<0.001
qPCR	78	20.2 (49; 17.4-22.9)	26.5 (29; 24.9-27.6)	79	<0.001
Microscopy[3]	71	20.3 (34; 17.3-23.0)	25.1 (37; 21.5-27.3)	28	<0.001
Culture	29	19.4 (13; 14.3-22.0)	23.8 (16; 17.7-25.5)	21	0.013
Includes samples with azithromycin (excludes phage samples)					
RDT	63	18.4 (22; 16.2-21.1)	23.9 (41; 21.3-26.2)	46	<0.001
qPCR	63	20.2 (43; 17.4-22.9)	26.2 (20; 24.5-27.5)	65	<0.001
Microscopy[3]	58	20.3 (32; 17.4-23.2)	24.5 (26; 20.7-26.2)	18	0.002
Culture	27	19.4 (13; 14.3-22.0)	23.8 (14; 17.8-25.5)	21	0.011
Includes samples with phage (excludes azithromycin samples)					
RDT	56	19.0 (23; 16.5-21.7)	24.6 (33; 21.1-27.0)	48	<0.001
qPCR	56	19.9 (41; 17.4-22.8)	27.2 (15; 25.4-27.6)	163	<0.001
Microscopy[3]	52	19.1 (27; 17.2-22.8)	24.8 (25; 21.5-27.2)	53	<0.001
Culture	22	19.4 (13; 14.3-22.0)	20.8 (09; 17.6-25.9)	2.8	0.186

[1] Nanoliter (nL) qPCR positive for *V. cholerae* (Ct < 28); a random subset were cultured or available for microscopy.
[2] Mann-Whitney U test (two-tailed).
[3] Indeterminant samples were scored as negative; limit of detection 100-1000 CFU/ ml.
[4] Difference in positive and negative Ct values with transformation from base-two to base-ten.

Table S4. Estimated sensitivity and specificity of theoretical diagnostic modalities

Diagnostic Modality[1]	Sensitivity (95% CI)	Specificity (95% Ci)
RDT alone	0.291 (0.19-0.395)	0.999 (0.996-1)
Culture alone	0.448 (0.276-0.63)	0.986 (0.968-1)
V. cholerae qPCR *(tcpA)* alone	0.629 (0.521-0.739)	0.997 (0.993-1)
ICP1 PCR alone	0.178 (0.101-0.274)	0.978 (0.967-0.988)
ICP1 PCR or RDT	0.463 (0.349-0.571)	0.978 (0.967-0.988)
ICP1 PCR or culture	0.646 (0.486-0.778)	0.923 (0.888-0.954)
ICP1 PCR or *V. cholerae* qPCR *(tcpA)*	0.743 (0.643-0.838)	0.938 (0.902-0.969)

[1] These estimates are graphically shown in Figure 1C and do not include latent class modeling.

References for Supplemental Materials

1. Alexandrova L, Haque F, Rodriguez P, Marrazzo AC, Grembi JA, Ramachandran V, Hryckowian AJ, Adams CM, Siddique MSA, Khan AI, Qadri F, Andrews JR, Rahman M, Spormann AM, Schoolnik GK, Chien A, Nelson EJ. 2019. Identification of widespread antibiotic exposure in cholera patients correlates with clinically relevant microbiota changes. J Infect Dis doi:10.1093/infdis/jiz299.
2. Mekalanos JJ. 1983. Duplication and amplification of toxin genes in *Vibrio cholerae*. Cell 35:253-63.
3. Rahman M, Jubair M, Alam MT, Weppelmann TA, Azarian T, Salemi M, Sakharuk IA, Rashid MH, Johnson JA, Yasmin M, Morris JG, Jr., Ali A. 2014. High-frequency rugose exopolysaccharide production by *Vibrio cholerae* strains isolated in Haiti. PLoS One 9:e112853.
4. Felsenfeld O, Stegherr-Barrios A, Aldova E, Holmes J, Parrott MW. 1970. In vitro and in vivo studies of streptomycin-dependent cholera vibrios. Appl Microbiol 19:463-9.
5. Hoshino K, Yamasaki S, Mukhopadhyay AK, Chakraborty S, Basu A, Bhattacharya SK, Nair GB, Shimada T, Takeda Y. 1998. Development and evaluation of a multiplex PCR assay for rapid detection of toxigenic *Vibrio cholerae* O1 and O139. FEMS Immunol Med Microbiol 20:201-7.
6. Nandi B, Nandy RK, Mukhopadhyay S, Nair GB, Shimada T, Ghose AC. 2000. Rapid method for species-specific identification of *Vibrio cholerae* using primers targeted to the gene of outer membrane protein OmpW. J Clin Microbiol 38:4145-51.
7. Grembi J, Mayer-Blackwell K, Luby S, Spormann A. 2019. High-throughput multi-parallel enteropathogen quantification via nano-liter qPCR. bioRxiv 746446; doi: https://doi.org/10.1101/746446 Accessed March 9, 2020.
8. Seed KD, Bodi KL, Kropinski AM, Ackermann HW, Calderwood SB, Qadri F, Camilli A. 2011. Evidence of a dominant lineage of *Vibrio cholerae*-specific lytic bacteriophages

shed by cholera patients over a 10-year period in Dhaka, Bangladesh. MBio 2:e00334-10.

9. Maeda H, Fujimoto C, Haruki Y, Maeda T, Kokeguchi S, Petelin M, Arai H, Tanimoto I, Nishimura F, Takashiba S. 2003. Quantitative real-time PCR using TaqMan and SYBR Green for *Actinobacillus actinomycetemcomitans*, *Porphyromonas gingivalis*, *Prevotella intermedia*, tetQ gene and total bacteria. FEMS Immunol Med Microbiol 39:81-6.

10. Chung WS, Walker AW, Louis P, Parkhill J, Vermeiren J, Bosscher D, Duncan SH, Flint HJ. 2016. Modulation of the human gut microbiota by dietary fibres occurs at the species level. BMC Biol 14:3.

11. Ontweka LN, Deng LO, Rauzier J, Debes AK, Tadesse F, Parker LA, Wamala JF, Bior BK, Lasuba M, But AB, Grandesso F, Jamet C, Cohuet S, Ciglenecki I, Serafini M, Sack DA, Quilici ML, Azman AS, Luquero FJ, Page AL. 2016. Cholera Rapid Test with Enrichment Step Has Diagnostic Performance Equivalent to Culture. PLoS One 11:e0168257.
